# Evaluation of sexual and gender-based violence program in Harare City, Zimbabwe, 2016: a descriptive cross-sectional study

**DOI:** 10.11604/pamj.2018.31.200.14791

**Published:** 2018-11-22

**Authors:** Zvanaka Sithole, Notion Tafara Gombe, Tsitsi Juru, Prosper Chonzi, Gerald Shambira, Peter Nsubuga, Mufuta Tshimanga

**Affiliations:** 1 MPH Programme, Department of Community Medicine, University of Zimbabwe, Zimbabwe; 2 City Health Directorate, Harare, Zimbabwe; 3 Global Public Health Solutions, Atlanta, Georgia, USA

**Keywords:** Sexual Gender-Based Violence, program, Harare city

## Abstract

**Introduction:**

In Zimbabwe, there is a gap between sexual violence (SV) survivors' health care needs versus the existing facilities. Harare city started Sexual Gender Based Violence (SGBV) project in 2011, with the aim to reduce SV morbidity.Only 592 (42%) of 1425 SV survivors reported for medical services within 72 hours in 2015. HIV post-exposure prophylaxis (PEP) is effective within 72hours of post exposure. We evaluated the program performance in Harare city.

**Methods:**

We conducted a process-outcome evaluation using a logic model. We purposively recruited all eight SGBV sites and key informants. We randomly selected 27nurses into the study. Interviewer-administered questionnaires and checklists were used to collect data. To generate frequencies, means and proportions we used Epi info 7.

**Results:**

The program adequately received inputs except for counselling rooms (1/8). About 4285 survivors were recorded from 2013-2016. Of these, 97% were counselled, 93% received HIV test, 41% reported to the clinic within 72hrs of post-rape, and 12% received PEP. About 16% of the total survivors were followed up. The programme failed to meet its targets on decentralised sites (8/10), awareness campaigns(16/32) and sensitisation activities(16/32). About 500(12.5%) IEC materials were distributed. All 96-targeted supervisory visits were achieved. Two ofeight district supervisors were trained. Majority of health workers (25/35) citedlack of awareness as major reasons for underperformance.

**Conclusion:**

Availability of resources did not translate to program performance. Most survivors were not reporting to the clinic timeously due to the low level of awareness of the programme to the community, hence were not protected from getting HIV through PEP. The programme was not well disseminated, as most supervisors were not trained. Following this evaluation, we distributed150 IEC materials to each of the eight facilities. A follow-up study on outcomes of clients referred for services and training of district officers were recommended.

## Introduction

Sexual violence (SV) refers to any act attempt or threat of a sexual nature that results or is likely to result, in physical, psychosocial and emotional harm [1]. Sexual violence is a gross violation of human rights and an issue of public health concern [2]. Survivors of sexual violence are more vulnerable to depression, substance abuse, repeat sexual abuse, Post Traumatic Stress Disorder (PTSD), suicide and sexual dysfunction later in life than their non-abused counterparts [3]. Globally, about 7% of women have been sexually assaulted by someone other than a partner [4]. Many UN organisations including the World Health Organization (WHO) argue that the deep-rooted and pervasive gender inequalities and sexual violence are responsible for the high and accelerated prevalence of HIV among women [5]. Women are at up to six times at greater risk of HIV infection compared to their male counterparts [6]. The gender ratio of infections reflects the greater vulnerability of women [7]. Sexual violence is a widespread problem in sub-Saharan Africa [8], and Sub-Saharan African countries are increasingly responding to sexual violence with a range of legislative and healthcare interventions [9]. Zimbabwe is not spared from the crimes of sexual violence. According to the 2015 Zimbabwe Demographic and Health Survey (ZDHS), 35% of all women, aged 15-49 reported having experienced physical violence [10]. In Zimbabwe, there is a gap between the healthcare needs of survivors of sexual violence and the existing level of health care provided, since most doctors and nurses have not received adequate training in the management of sexual violence. Specialist clinics were in central hospitals but were not easily accessible to the majority of sexual violence survivors who needed prompt and appropriate management close to their homes [11]. In light of the health, human rights, social and economic consequences of violence against women, Harare City in collaboration with Medicines San Frontiers (MSF) started a separate Sexual Gender Based Violence (SGBV) project in 2011 with the aim to reduce the morbidity and mortality of survivors of sexual violence in Harare city [12]. In Harare city, the SGBV program follows the Ministry of Health and Child Care (MOHCC) national protocol on care for survivors of SGBV. It offers free medical care, HIV testing and counselling, screening and referrals for psychological, psychosocial and legal support. According to the Harare city data, sexual violence increased from 1162 in 2014 to 1356 survivors in 2015. Of concern is that only 42% of the survivors attended to in 2015 reported for medical services within 72 hours. It is a cause for concern considering that HIV post-exposure prophylaxis (PEP) is effective within 72hours of post exposure. In the light of these issues, we here consider the question "why is the 58% of the SV survivors not accessing quality post-rape services promptly?" We therefore broadly evaluated the performance of the programme and specifically assessed the inputs, processes, outputs and outcomes of the program.

## Methods

**Study design:** We conducted a process-outcome evaluation using the logic model. This was used to assess the inputs, which were injected into the program, the processes carried out, the outputs realised and the outcomes of the SGBV program (Table 1).

**Table 1:** Logic model for the SGBV program in Harare city, 2016

Inputs	Activities	Outputs	Outcomes	Impact
Human resources Pharmaceutical & Laboratory supplies Policy guidelines Training manuals Computers Activity log book Tally sheets IEC materials Patient files registers Stationery Network of service providers Drama group Financial Resources Transport	Training of staff. Workshops. Stakeholder meetings. Provision of treatment and care. Raising awareness. Follow up presentation. Referring to social and legal support. Referring for Termination Of Pregnancy(TOP). Decentralization of the SBGV services. Drama performance. Organizing income generating activities. Support and supervision	Number of trained staff Number of meetings held. Number of survivors that received treatment. Number of new survivors coming to the clinic. Number of survivors that were followed up. Number of survivors referred for social and legal support. Number of referred survivors that had TOP. Number of survivors of SGBV attended to. Stand-alone sites.	Improved quality of care % reduction in STIs % reduction in late presentations (>72hrs) % reduction in psychosocial disorders Multi-sectoral approach Increased access to comprehensive medical package	Reduced morbidity and mortality related to SGBV

**Input indicators:** Inputs are the resources and materials invested into the program. These include staff, money, vehicles, laboratories and laboratory equipment, health facilities offering services, drugs, treatment and policy guidelines, SGBV manuals, information education communication materials and case definitions.

**Process indicators:** Processes were the activities conducted utilising the inputs provided for the program. These included staff training, workshops, meetings, referring survivors for social, psychological and legal services, treatment, organising income generating activities and supportive supervision.

**Output indicators:** These were the short-term logical results of implementing program activities. These included number of survivors reporting to the stand-alone sites, number of health workers trained, number of meetings held, number of survivors referred for services, number of workshops held and number of survivors followed up.

**Outcome indicators:** Programme outcomes are medium-term logical consequences/results of achieving a number of outputs. These include improved quality of care, thepercentage reduction in STIs, percentage reduction in late presentations (> 72hrs) percentage reduction in psychosocial disorders and Increased access to thecomprehensive medical package.

**Study setting:** We conducted the study at Harare City SGBV clinics.

**Study population:** Nurses in the facilities that offer SGBV services were recruited into the study. The HIV Program Manager, the District Medical Director (DMO) and the Reproductive Health Program Manager were recruited as key informants.

Sample size calculation: Using Dobson formula:

$$n=Z_{a}^{2}\left(p\right)\left(1-p\right)/delta^{2}$$

Where Z_2_ = 1.96, p = 0.021(Gender links (2010) in Gauteng, South Africa [**13**], with proportion of sexually abused women who reported the case being 2 %), delta = 0.05, confidence interval = 95%, non-response rate is 10%, a minimum sample size of 32 health workers was calculated.

**Sampling procedure:** SGBV program Coordinator, HIV and opportunistic infections and antiretroviral therapy (OI/ART) focal persons, district medical officers (DMO), district nursing officers (DNO), reproductive health program manager and HIV program manager were purposively recruited into the study as key informants. All nurses who participated in the program in the district were includedin the study, except those who were on leave.

**Pretesting:** The data collection instruments were pretested at Mbare polyclinic for validity and reliability. Ambiguous questions were corrected accordingly.

**Data collection:** We used an observational checklist to check for availability of inputs for the program including health-worker training register, register for SGBV survivors in the program, SGBV program stationary, and SGBV policy guidelines, budget requests, reports on SGBV awareness coverage for the district. To assess the output and outcome indicators as well as resource availability we conducted records review. Interviewer-administered questionnaires were used to elicit information on operations of the program, reasons why survivors are not accessing quality post-rape services promptly from study participants. Key informant interview guide was used to gather information about the number of health workers trained in SGBV management. The checklist structured from US Centers for Disease Control and Prevention (CDC) guidelines on programme evaluation, were used to quantify the resources required to run the programme [14].

**Data analysis:** We used Epi-info version 7 to generate frequencies and means. Qualitative data generated from open-ended questions were sorted and analysed for content. Summaries of this data were presented as frequencies and proportions of responses.

**Permission and ethical considerations:** We sought permission to carry out the study from Harare city ethical review board, and the Health Studies Office. We also sought written informed consent from the health workers. Participants were given the freedom of choiceto either participate, decline or withdraw from the study at any given time. Confidentiality was assured and maintained throughout the study by; interviewing each participant privately and ensuring that no information obtained was disclosed to any persons other than those relevant to the study. Also, names of participants were not includedinthe questionnaires. All questionnaires were kept confidential.

## Results

**Demographic characteristics of study participants:** We interviewed thirty-five study participants with the majority being females 91% (32/35). The majority 27(77.1%) were nurses offering SGBV services in Harare City. The median years of service were 11 years (Q1 = 10.5; Q_3_= 15). The median age was 36 years (Q_1_= 32; Q_3_=40).

**Inputs Injected into the SGBV Program:** The program adequately received targeted resources as far as guidelines, IEC materials registers, HIV test kits and fuel were concerned. The program had one of eight targeted rooms for counselling of the SV survivors (Table 2).

**Table 2:** Inputs injected into Sexual Gender Based Violence Program, Harare City, 2016

Inputs	Achieved	Target/	% Achieved
Treatment guidelines	1	1	100
IEC materials	4000	4000	100
Telecommunication system	1	1	100
Fuel	11520	11520	100
HIV rapid test kits	1000	100	100
HIV PEP	60	60	100
Pregnancy ECP	60	60	100
STI PEP	120	120	100
Registers	1	1	100
Affidavit book	1	1	100
Vehicles	4	4	100
Counselling rooms	1	8	12.5

**Processes involved in running the sexual gender based violence programme:** Two targeted staff training courses per yearwere met. About 16% (700/4285) of clients were followed up. The programme failed to meet its targets on decentralisation of SGBV sites (8/10), awareness campaigns (16/32) and sensitisation activities (16/32) (Table 3).

**Table 3:** Processes involved in running the Sexual Gender Based Violence programme, Harare City, 2016

Process	Target	Achieved	% Achieved
Staff trainings	2	2	100
Decentralisation	10	8	80
Planning	4	4	100
Budgeting	1	1	100
Stakeholder and community sensitisation activities	32	16	50
Distribution of IEC materials			
Awareness campaigns	32	16	50
Support and supervisory visits	96	96	100
Client follow up activities	4285	700	16

**Outputs of the sexual gender based violence programme:** The programme did not meet the targeted number of health care workers trained in SGBV program (80/100). The number of clients followed up (700/4285), and clients reported with 72 hours (1758/ 4285) and received PEP post-sexual violence (1758/1213) were below the target. The targeted number of IEC materials to be distributed (500/4000), monthly supportive and supervisory visits (96/96) and clients who received STI PEP (2770/2770) were achieved (Table 4).

**Table 4:** Outputs of the sexual gender based violence programme, Harare City, 2016

Indicators	Target	Achieved	% Achieved
Number. of health workers trained	100	80	80
Number. of IEC materials distributed	4000	500	12.5
Number of clients reported<72hrs	4285	1758	41
Number of clients received HIV PEP	1758	1213	69
Number of clients received HIV testing	4285	3970	93
Number of clients received STI PEP	2770	2770	100
Number of clients received counselling	4285	4170	97
Number of support &supervisory visits done	96	96	100
Number of clients followed up	4285	700	16
Number of clients tested HIV positive	0	163	-

**Outcomes of the sexual gender based violence programme:** From January 2013 to December 2016, there has been an increase in the number of SV survivors who reported to the clinic timeously (< 72hours), from zero to 761 cases. Proportions of SV survivors who received PEP increased again from zero out of three in 2013 to 509 (31%) out of 1669 in 2016. Those who received counselling increased from 33% in 2013 to 65% in 2016 (Table 5).

**Table 5:** Sexual gender based violence outcomes, Harare city, 2011-2016

Indicator	2013 N=3 N (%)	2014 N=1188 N (%)	2015 N=1425 N (%)	2016 N=1669 N (%)	% Increase
Proportion reported timeously (<72 hours)	0 (0)	413 (35)	592 (42)	761 (46)	46%
Proportion receiving HIV PEP	0 (0)	293 (25)	410 (29)	509 (31)	31%
Proportion received counselling	1 (33)	1136 (96)	1396 (98)	1635 (98)	65%
Proportion acquired HIV	3 (100)	17 (3)	62 (4.4)	60 (3.6)	96.4%
Proportion pregnancy ECP	1 (33)	242 (20)	439 (31)	424 (25)	8%
Proportion received STI PEP	1 (33)	816 (167)	967 (68))	986 (59)	26%

**Knowledge levels of health workers on sexual gender based violence, Harare city, 2016:** There were good and fair knowledge levels among nurses and district officers respectively. All 35 respondents (100%) knew the main objectives of the SGBV program. One (25%) of four doctors knew the tools used to monitor the program. All nurses trained on management of SV survivors were knowledgeable in all areas assessed. One (25%) of four district nursing officers knew the correct standard treatment guidelines for the management of SV survivors. All doctors (100) knew the standard guidelines for management of sexual violence survivors. However, three (75%) of them, did not know the management process for sexual violence survivors.

**Reasons for late reporting to the hospital by sexual violence survivors:** Information on reasons for late reporting was extracted from the SV register. The health workers were also asked on the reasons for theunderperformance of the program. Out of 1669 SV survivors in 2016, a total of 908 (54%) did not report timeously to the hospital for quality services. The main reasons recorded in the registers were survivor not aware of the health facilities that offer post-rape services 398 (44%),late disclosure of the survivor to the guardian or partner 202 (22%), no bus fare 167(18%), fear of being harmed further by the perpetrator 97(11%). Of the 35 health workers interviewed Majority 25 (71%) cited the lack of awareness of the program as a major reason for underperformance.

## Discussion

The study findings demonstrate that lack of awareness of the program and its services was a major factor that enhances thelate presentation of SV survivors to the clinic. Closely related to the late reporting was fear of being harmed further by the perpetrator manifested especially in thecontext of late disclosure of the survivor to the guardian or partner. This study sought to answer "why the SV survivors were not accessing quality post-rape services promptly?" Therefore, we evaluated the performance of the SGBV programme. It is evident from the study that the program was not well disseminated as no district officers were trained on SGBV. This finding maybe due to lack of integrated planning between donors and Harare city senior management. The lack of training among district officers explains why the supportive visits were not being conducted as targeted. This finding threatens the sustainability of the program in the city. The adequate funding injected into the program did not translate to the good performance of the program. Contrary to the findings by Vetten 2007, which showed SGBV programs lack specific budgets, resulting in implementation problems, this program was adequately funded [15]. However, although the IEC materials were adequately injected to the program, they were not distributed to the stand-alone sites. A low proportion of the survivors that reported to the clinic within 72 hours reduced proportions getting pregnancy emergency contraceptive prevention (ECP) and the unmet IEC materials distributed suggest a low level of awareness of the program to the community. The low proportion of the survivors that reported to the clinic within 72 hours shows that most survivors are not being protected from getting HIV as PEP is effective within 72 hours of post exposure. According to Savage *et al*. 2015, South Africa,unwanted pregnancies could be prevented by ensuring adolescents have access to information on sexuality and are supported to build good social and decision-making skills in a supportive environment [9]. In this study, SGBV information and support were not fully offered. The low level of the programme awareness to the community may show that there may be other SV cases that are not being reported since survivors would not be aware of services available for them. Ferguson (2009) highlighted that the victim of sexual assault rarely reports the crime to police [16, 17]. It is, therefore,the role of healthworkers to raise awareness on sexual violence [18].

The low level of the programme awareness may suggest under-estimation of the prevalence of sexual violence in Harare. We also found out that the program did not achieve its targeted decentralised health facilities. This might be explained by the unmet targeted number of health care workers trained in SGBV program. For the program to come up with stand-alone sites, human resources would be needed. Our study suggests that the low proportions of clients that received pregnancy ECP might have been due to the sending back of clients who would have reported to the clinic during busy hours of the clinic as reported by the nurses. These survivors would be advised to report after four days or so, due to overwhelming workload. This actis not ethical according to the WHO guidelines on themanagement of sexual survivors [6]. This act also reflects that some of the survivors are not receiving quality post-rape services in Harare city. Although the proportions of survivors who received counselling were high, the counselling sessions were being done hurriedly, hence compromising quality. On investigation, the counselling sessions were conducted hurriedly due to the use of multi-purpose rooms for counselling sessions with no privacy. These findings are contrary to other studies that highlight the need for safe access to Sexual and Reproductive Health services like the right to consent to services that are confidential, and private [19, 20]. Although ZDHS is showing some positive results in the area of Gender Based Violence (GBV) in Zimbabwe (sexual violence prevalence decreasing from 18.4% in 2010 to 14.5% in 2015) [8], cases of SGBV in Harare were increasing with years. The difference between the national and Harare city sexual violence trends reflects the higher magnitude of the problem in Harare urban as compared to others. Our study provides evidence that training of health care workers HCW is essential. These findings extend the study done among victims of Family Support Trust units in Harare, 2010, which showed a strong association between exposure to training on sexual abuse issues and knowledge of guidelines and standards for supporting child survivors [14]. The proper management of cases regarding treatment in this study might be due to the highly knowledgeable trained SGBV nurses and availability of treatment guidelines. Our study had some limitations. No tracking was done to check if the referred clients received the intended services. However, a follow-up study on referred clients was recommended. We did not interview the survivors to determine the reasons for late presentation. However, some reasons were extracted from the survivors' records.

## Conclusion

We concluded that even though this programme was adequately funded, it was not well disseminated. There is no joint venture in planning and implementation of the program between the Harare city district supervisors and MSF, hence planning and integration was lacking. District supervisors were not actively involved in the supervisory activities of this program. Nurses had higher program knowledge as compared to their supervisors. There is still low level of awareness of the program to the community. Based on the findings of this study, a follow-upstudy to check if the referred clients received the intended services were recommended. The study recommends training of district officers on themanagement of SV survivors so that they can have the capacity to conduct the supportive supervisory activities.Integrated planning between donors and senior management as a foundation for successful program implementation was also recommended. The public health officer facilitated the distribution of the IEC materials to all stand-alone sites.

### What is known about this topic

- Sexual violence against women is a massive cause of morbidity and mortality but remains overlooked. While rape will always be a traumatic experience and a violation of human rights, the effects of this trauma for an individual may be different in different contexts. Hence quality care should be rendered to these survivors.

### What this study adds

- Availability of resources does not translate to program performance. Therefore, inputs need to be converted to outputs through activities.Programs need to be well disseminated for them to be sustainable. Knowledgeable workers are key to the implementation of the programme. Hence there is need to train HCW before the program starts.

## Competing interests

The authors declare no competing interests.
